# Natural alleles of *GLA* for grain length and awn development were differently domesticated in rice subspecies *japonica* and *indica*

**DOI:** 10.1111/pbi.13080

**Published:** 2019-03-09

**Authors:** Yanpei Zhang, Zhanying Zhang, Xingming Sun, Xiaoyang Zhu, Ben Li, Jinjie Li, Haifeng Guo, Chao Chen, Yinghua Pan, Yuntao Liang, Zhijian Xu, Hongliang Zhang, Zichao Li

**Affiliations:** ^1^ Key Laboratory of Crop Heterosis and Utilization Ministry of Education/Beijing Key Laboratory of Crop Genetic Improvement Department of Plant Genetics and Breeding China Agricultural University Beijing China; ^2^ China/Guangxi Key Laboratory of Rice Genetics and Breeding Rice Research Institute Guangxi Academy of Agricultural Sciences Nanning Guangxi China

**Keywords:** allelic frequency, awn development, domestication, grain length, natural variation

## Abstract

Rice (*Oryza sativa* L.) cultivars harbour morphological and physiological traits different from those of wild rice (*O. rufipogon* Griff.), but the molecular mechanisms underlying domestication remain controversial. Here, we show that awn and long grain traits in the near‐isogenic NIL‐GLA are separately controlled by variations within the *GLA* (*Grain Length and Awn Development*) gene, a new allele of *GAD1*/*RAE2*, which encodes one member of the EFPL (epidermal patterning factor‐like protein) family. Haplotype analyses and transgenic studies revealed that InDel1 (variation for grain length, VGL) in the promoter region of *GLA* ( *GLA* ^*VGL*^ ) increases grain length by promoting transcription of *GLA*. Absence of InDel3 (variation for awn formation, VA) in the coding region (CDS) of *GLA* (*GLA* ^*va*^) results in short awn or no awn phenotypes. Analyses of minimum spanning trees and introgression regions demonstrated that *An‐1*, an important gene for awn formation, was preferentially domesticated and its mutation to *an‐1* was followed by *GLA* and *An‐2*. Gene flow then occurred between the evolved *japonica* and *indica* populations. Quality analysis showed that *GLA* causes poor grain quality. During genetic improvement, awnlessness was selected in ssp. *indica*, whereas short–grained and awnless phenotypes with good quality were selected in *japonica*. Our findings facilitate an understanding of rice domestication and provide a favourable allele for rice breeding.

## Introduction

Rice (*Oryza sativa* L.), one of the earliest domesticated, primary food crops, feeds more than half of the world population (Khush, 1997). It originated from wild rice (*O. rufipogon* Griff.) through domestication (Fuller *et al*., 2010; Huang *et al*., 2012; Zong *et al*., 2007). Wild rice has unfavourable agronomic traits, such as prostrate growth, seed shattering, a spreading panicle, long awns and low number of grains per panicle, all of which changed dramatically under domestication. Compared with its wild ancestors cultivated rice typically exhibits reduced seed shattering and dormancy, increased grain number per panicle, erect growth, closed panicles and no or short awns (Kovach *et al*., 2007; Sweeney and McCouch, 2007). All of these features that differ between wild rice and cultivars are known as domestication traits.

To date, some domesticated genes are functionally characterized, such as seed shattering genes *Sh4* (Li *et al*., 2006) and *qSH1* (Konishi *et al*., 2006; Li *et al*., 2006), *prog1* controlling straight growth (Jin *et al*., 2008; Tan *et al*., 2008), *OsLG1* regulating closed panicles (Ishii *et al*., 2013; Zhu *et al*., 2013), grain size genes *GW5/GSE5* (Duan *et al*., 2017; Liu *et al*., 2017), *GS3* (Mao *et al*., 2010), *OsLG3* (Yu *et al*., 2017) and *OsLG3b* (Yu *et al*., 2018), seed dormancy gene *Sdr4* (Sugimoto *et al*., 2010), seed hull colour genes *Bh4* (Zhu *et al*., 2011) and *Rc* (Sweeney *et al*., 2006), *NOG1* increasing grain production (Huo *et al*., 2017) and awn development genes *An‐1* (Luo *et al*., 2013), *An‐2/LABA1* (Gu *et al*., 2015; Hua *et al*., 2015) and *GAD1/RAE2* (Bessho‐Uehara *et al*., 2016; Jin *et al*., 2016).

Awn, one of the most important domestication traits in cereal crops, is common in graminaceous crops such as sorghum (*Sorghum bicolor* L.), wheat (*Triticum aestivum* L.), oat (*Avena sativa* L.) and barley (*Hordeum vulgare* L.). Long awns, extensions at the tip of the lemmas, help seed dispersal and prevent birds and mammals from preying on the grains (Elbaum *et al*., 2007; Hu *et al*., 2011; Kulic *et al*., 2009). In some cereal crops, such as wheat and barley, awns also have photosynthetic capability that contributes to grain yield (Abebe *et al*., 2010). However, rice awns have no chloroplasts and are inconvenient for harvesting and post‐harvest processing (Takahashi *et al*., 1986). Hence, domesticated rice usually possesses no or short awns (Toriba *et al*., 2010).

The awn is a complex character controlled by multiple genes. Several awn‐related genes in rice have been cloned. *An‐1*, encoding a bHLH transcription factor, positively controls the formation of awn primordia, cell division and grain length in wild rice. The *an‐1* allele conferring shorter awns, shorter grains and higher number of grains per panicle was selected during domestication (Luo *et al*., 2013). *An‐2*/*LABA1* encodes a cytokinin synthesis enzyme and increases the cytokinin concentration in awn primordia, thus promoting awn elongation. It also negatively regulates grain number per panicle and tiller number per plant (Gu *et al*., 2015; Hua *et al*., 2015). *RAE2*/*GAD1* encodes a secreted signal protein member of the epidermal patterning factor‐like family. The precursor peptide of *RAE2*/*GAD1* must be ruptured under the action of lyase to form a mature polypeptide. *RAE2*/*GAD1* regulates awn development, as well as grain number per panicle and grain length (Bessho‐Uehara *et al*., 2016; Jin *et al*., 2016). The above reports demonstrated that genes controlling awn phenotype have pleiotropic effects on yield‐related traits. Long awn and grain length are often closely associated, suggesting that alleles conferring longer grain might be neglected because of the selective sweep of awn traits. Currently, the relationship between these traits is not clear and the genetic basis and molecular mechanisms conferring the selective forces involved in awn formation and grain length during domestication need to be further examined.

Here, we isolated a multifunctional gene *GLA* (*Grain Length and Awn Development*), which is an allele of *GAD1/RAE2*. Using *GLA*‐gene association analysis, haplotype analysis and transgenic studies, we analysed the functional variations of *GLA* for grain length and awn formation. Evolutionary studies explained the relationship among the *An‐1*,* An‐2* and *GLA* genes during the domestication process. In addition, quality evaluation and allele utilization were used to analyse the selection histories of *japonica* and *indica* rice in the process of genetic improvement. These studies not only improve our understanding of crop domestication, but also provide a valuable genetic resource for future molecular breeding of rice.

## Results

### Phenotypic characterization of NIL‐GLA and NIL‐gla

**Figure 1:**
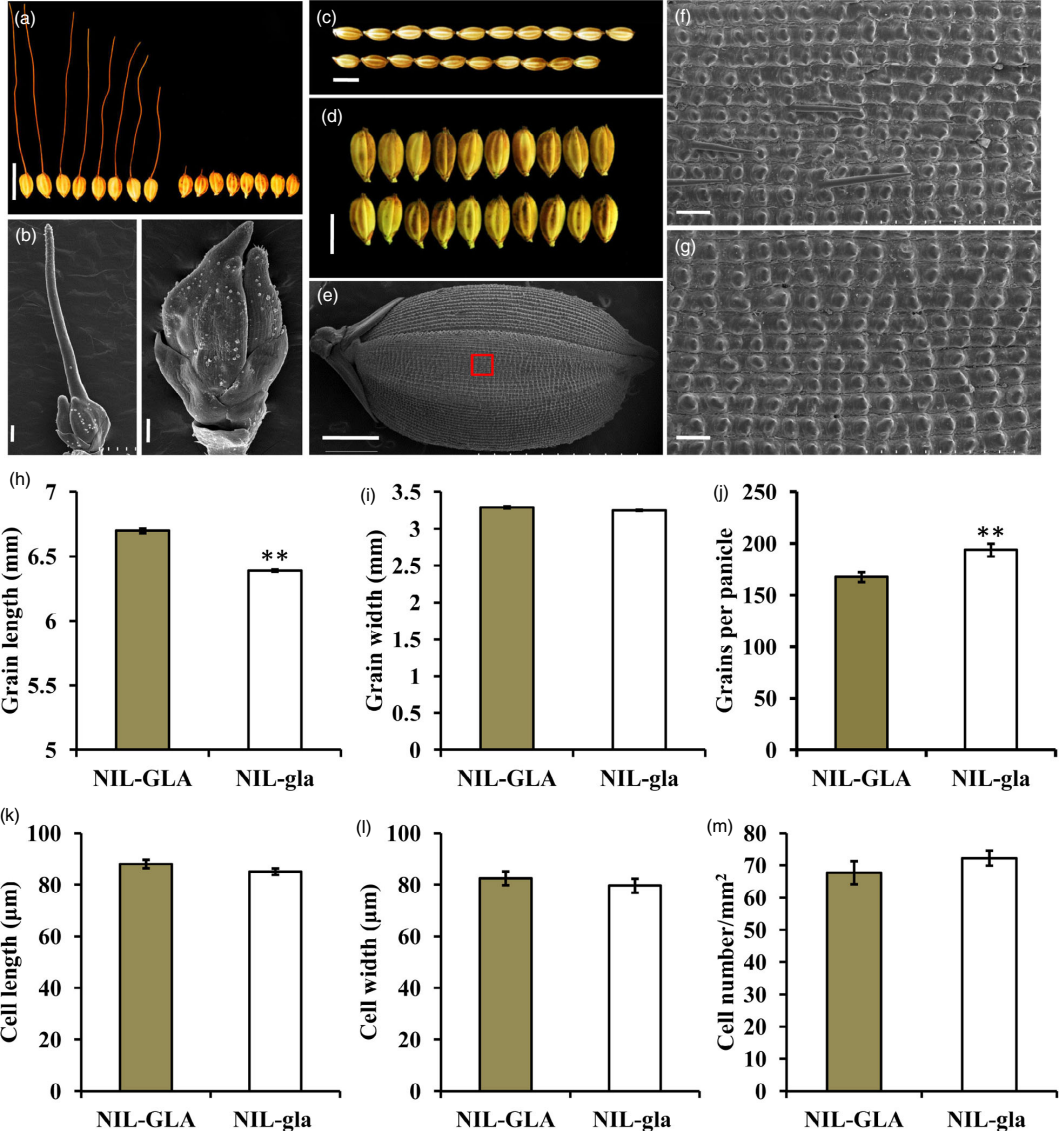
Phenotypes of NIL‐GLA and NIL‐gla NIL plants. (a) Awn phenotypes of NIL‐GLA (left) and NIL‐gla (right). Bar, 10 mm. (b) SEM images of NIL‐GLA (left) and NIL‐gla (right) spikelets at the Sp8 l growth stage. Bar, 200 μm. (c,d) and (h,i) Comparison of grain length (c,h) and width (d,i) between NIL‐GLA (upper) and NIL‐gla (lower) NILs. Bar, 5 mm. (e) SEM image of a whole grain. Bar, 1 mm. The red boxed region is enlarged in SEM images of the outer epidermal cells of the lemma shown in NIL‐GLA (f) and NIL‐gla (g) grains. Bar, 100 μm. (j) Comparison of grain numbers per panicle between NIL‐GLA and NIL‐gla. (k–m) Comparisons of average cell length (k), average cell width (l) and average cell number per mm^2^ (m) on the outer surface of the lemmas of NIL‐GLA and NIL‐gla grains. Data are represented as means ± SE (*n *=* *15), **P <* *0.01 based on Student's *t*‐tests.

The backcross populations segregated at a single locus for the awn trait (Table S1). Long awns and long grains were completely associated in the BC_3_F_4_ population (Figure S1). The NIL‐GLA line with long awns and grains and NIL‐gla line with no awn and short grains were selected from the BC_3_F_4_ population (Figure S2). SEM of spikelet development at the Sp8I growth stage (Itoh *et al*., 2005) showed that the awn primordia of NIL‐GLA spikelets were more developed than in NIL‐gla spikelets (Figure 1a,b). NIL‐gla also developed much shorter grains and more grains per panicle than NIL‐GLA, but there was no significant difference in grain width (Figure 1c,d,h–j).

As awn development and grain elongation are related to cell division (Hua *et al*., 2015; Jin *et al*., 2016; Luo *et al*., 2013) the outer lemma surfaces of grains were examined using SEM (Figure 1e). There was no significant difference in average cell number per unit area, cell length or cell width between NIL‐GLA and NIL‐gla, suggesting that cell number rather than cell size was mainly responsible for the longer grains in NIL‐GLA (Figure 1f,g,k–m). These results demonstrated that the gene controlling awn phenotype in NIL‐GLA had pleiotropic effects on grain length and grain number per panicle. It was named *GLA* (*Grain Length and Awn Development*).

### *GLA* is a new allele at the *RAE2/GAD1* locus

**Figure 2:**
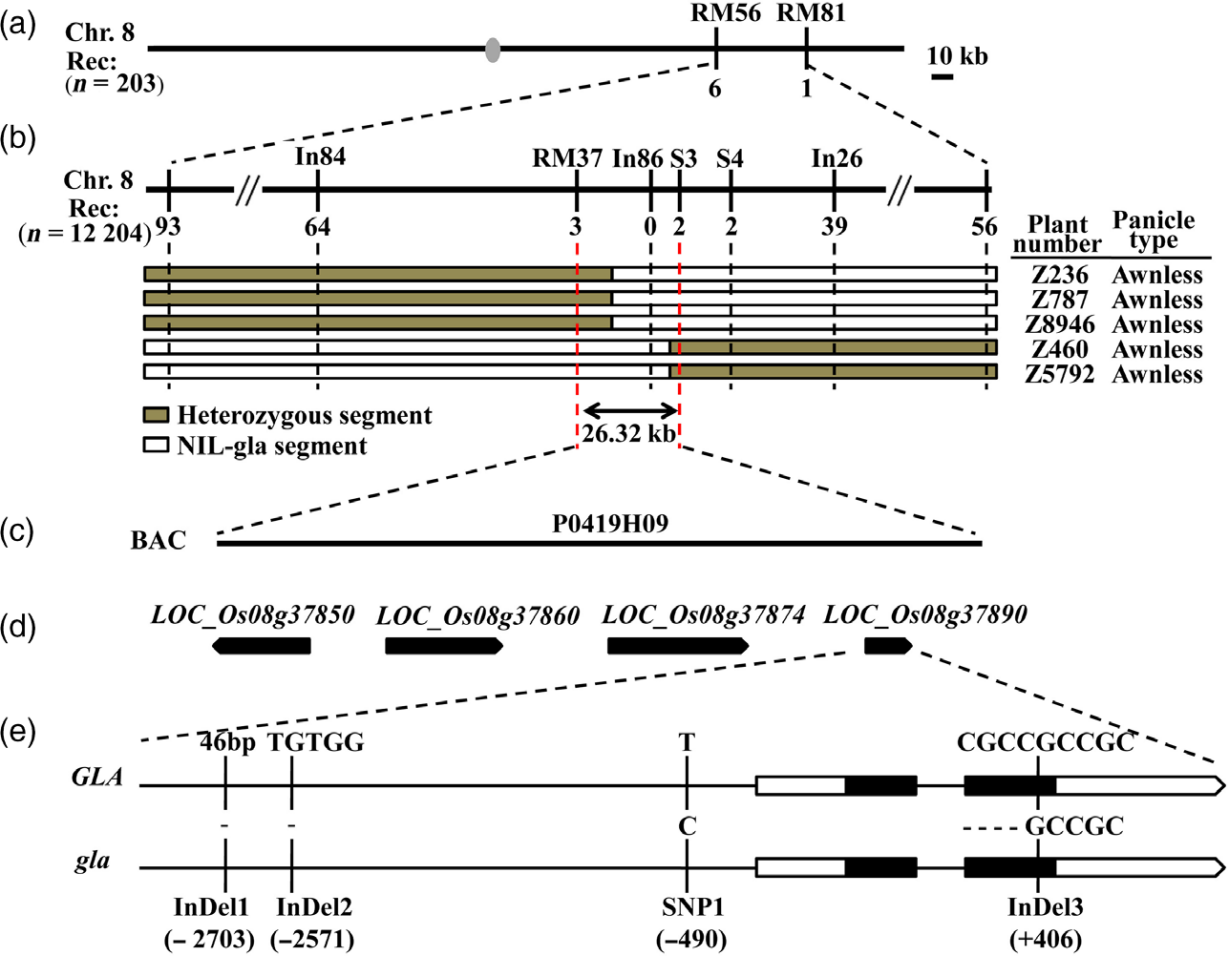
Fine mapping of *GLA*. (a) *GLA* was located between markers RM56 and RM81 on rice chromosome 8. Numbers of recombinants between *GLA* and molecular markers are shown below the bars. Grey ellipse represents the centromere. (b) *GLA* was fine mapped to a 26.32 kb region between markers RM37 and S3. Numbers of recombinants are given below the markers. Chromosome composition and panicle type of five recombinant plants are shown. White and grey bars represent chromosomal segment of NIL‐gla and heterozygotes respectively. (c) Nipponbare genomic BAC (P0419H09) containing this region. (d) Gene prediction by the Rice Annotation Project Database (http://rice.plantbiology.msu.edu/index.shtml). (e) Sequence variations between *GLA* in NIL‐GLA and *gla* in NIL‐gla. Black lines represent 5′ upstream regions and introns. White rectangles represent 5′ and 3′ UTR regions. Black rectangles represent coding regions. One SNP (SNP1) and three InDels (InDel1, InDel2 and InDel3) along with their positions were identified.

To isolate *GLA* 477 SSR markers were subjected to BSA (Zhang *et al*., 1994). Using 203 BC_3_F_3_ individuals for preliminary analysis, *GLA* was mapped between SSR markers RM56 and RM81 (Data S1) on the long arm of chromosome 8 (Figure 2a). Next, one SSR marker (RM37) and five STS markers (In84, In86, In26, S3, S4; Data S1) were used for fine mapping. Among 12 204 BC_3_F_4_ individuals, 2981 were awnless and *GLA* was delimited to a 26.32 kb genomic region between markers RM37 and S3 (Figure 2b).

Four genes (*LOC_Os08 g37850*,* LOC_Os08 g37860*,* LOC_Os08 g37874* and *LOC_Os08 g37890*) on BAC P0419H09 from this region were predicted by the Rice Annotation Project Database (http://rice.plantbiology.msu.edu/index.shtml) (Figure 2c,d, Table S2). Sequence analyses showed that differences between NIL‐GLA and NIL‐gla were present only in *LOC_Os08 g37890*; there were four variations, including three InDels and one SNP, namely, a 46 bp deletion (−2703, AGGTGTGGCATGGCAAACAAAGTGTGGCCAACAAATTGTTGGCCAC, InDel1), a 5 bp deletion (−2571, TGTGG, InDel2), a nucleotide substitution (−490, from T to C, named SNP1) in the promoter and a 4 bp nucleotide deletion (+406, CGCC, InDel3) in the second exon (Figures 2e and S3). Amino acid sequence analysis indicated that the InDel3 deletion caused a premature stop codon and truncated protein (Figure S4). *LOC_Os08 g37890* is the same locus as *Hap.A/B/C* (Yano *et al*., 2016), *GAD1* (Jin *et al*., 2016) and *RAE2* (Bessho‐Uehara *et al*., 2016; Jin *et al*., 2016; Yano *et al*., 2016) that belong to the epidermal patterning factor‐like protein (EPFL) family and regulate awn development. Thus, *LOC_Os08 g37890* was postulated to be the most likely candidate gene for *GLA*. Amino acid sequence alignments showed that *GLA* was a new allele of *RAE2/GAD1* (Figure S5).

In the previous studies, *GLA* encoded a pre‐propeptide with an N‐terminal signal peptide and a C‐terminal mature peptide that is specifically cleaved by SLP1 in rice (Bessho‐Uehara *et al*., 2016; Jin *et al*., 2016). However, the sub‐cellular localization of GLA remains unknown. Interestingly, we found that fluorescent signals of both GLA‐GFP and gla‐GFP fusion proteins could be detected in the cell membrane, cytoplasm and nuclei of rice protoplasts (Figure S6). It may be related to SLP1 cleavage in rice protoplasts. In addition, although *gla* encoded a truncated protein, there was no effect on sub‐cellular localization (Figure S6).

### Grain length and awn development are regulated by different functional variations of *GLA*

**Figure 3:**
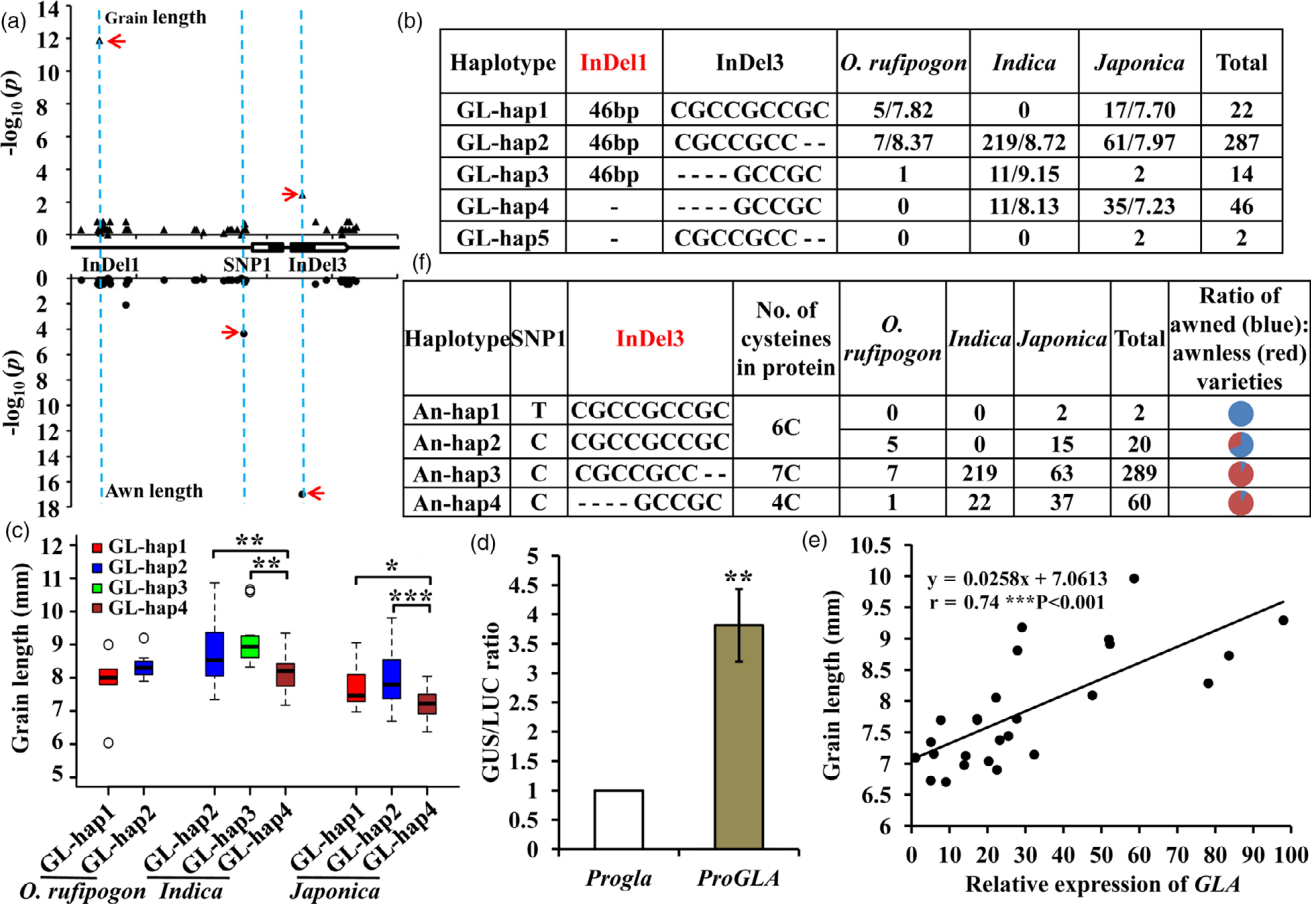
Variations in *GLA* were significantly associated with grain length and awn development. (a) Gene‐based association analysis of *GLA* for grain length (upper) and awns (lower) using a naïve model (*t*‐test; *P *=* *2.17 × 10^−2^). Red arrows represent significant loci. (b,f) Haplotype analysis of *GLA* for grain length (b) and awns (f). No., number. Average grain lengths of rice varieties harbouring corresponding haplotypes are indicated following the slash. (c) Comparison of grain lengths in different haplotypes. (d) Transient expression assays of promoter activity in tobacco leaves. *ProGLA* represents the presence of InDel1 in the promoter. *Progla* represents the deletion of InDel1 in the promoter. Each experiment consisted of three biological repeats with four technical replications. (e) Correlation analysis between *GLA* mRNA levels and grain length in 26 rice varieties assessed at Sanya (Hainan province).

*GLA* was shown to affect both grain length and awn traits and the functional loci for each trait were still not clear. *GLA*‐based association analyses were performed on the basis of 33 SNPs and 13 InDels in 358 cultivated accessions (Data S2). The results indicated that grain length and awn development were significantly associated with different variations. InDel1 and InDel3 made significant contributions to grain length, whereas SNP1 and InDel3 were significantly associated with awn formation (Figure 3a).

According to the significant variation associated with grain length, the sequences of 371 accessions including 358 cultivated and 13 wild rice varieties (Data S2) were divided into five haplotypes named GL‐hap1 to GL‐hap5. The wild rice accessions were mainly GL‐hap1 and GL‐hap2 types, *indica* accessions were GL‐hap2, GL‐hap3 and GL‐hap4 and *japonica* accessions were GL‐hap1, GL‐hap2 and GL‐hap4 (Figure 3b, Data S2). There was no significant difference in grain length between GL‐hap1 and GL‐hap2 in *O. rufipogon* and *japonica* or between GL‐hap2 and GL‐hap3 in *indica*. However, compared to GL‐hap1, GL‐hap2 or GL‐hap3, grain length of GL‐hap4 accessions was significantly reduced in both *japonica* and *indica* (Figure 3c, Data S2). These results suggested that grain length was influenced by the InDel1 (variation for grain length, VGL) in *GLA* (*GLA* ^*VGL*^).

Transient assays were performed in tobacco leaves to determine whether the promoter activity was influenced by *GLA* ^*VGL*^, which is located in the promoter. Relative promoter activities were significantly increased in *ProGLA* transformants containing the functional InDel1 (Figure 3d) suggesting that InDel1 increased the promoter activity of *GLA*. Moreover, transcriptional levels of *GLA* were positively correlated with grain length in 26 rice varieties randomly selected from the 358 cultivated varieties (Figure 3e, Data S3). These results indicated that *GLA* ^*VGL*^ regulated grain length by affecting the expression of *GLA*.

To determine the functional variation regulating awn formation on the basis of significant SNP/InDels, we classified the sequences of rice accessions into haplotypes An‐hap1, An‐hap2, An‐hap3 and An‐hap4. Comparing the numbers of cysteines (C) in GLA (Bessho‐Uehara *et al*., 2016) it became evident that An‐hap1 and An‐hap2 were 6C type, whereas An‐hap3 and An‐hap4 were 7C and 4C types respectively. The rare SNP1 variant in An‐hap1 occurred only in *japonica*. The InDel3 locus had three haplotypes. Compared with varieties of An‐hap2 (five awned varieties in *O. rufipogon* and nine awned and six awnless varieties in *japonica*), most varieties with An‐hap3 (54 of 63 varieties in *japonica*, 214 of 219 varieties in *indica*) and An‐hap4 (33 of 37 varieties in *japonica*, 22 varieties in *indica*) were awnless (Figure 3f, Data S2). This provided further evidence that InDel3 (variation for awn, VA) in *GLA* (*GLA* ^*VA*^) confirmed the functional variation in awn development, a result that is consistent with the previous results for *GAD1* (Bessho‐Uehara *et al*., 2016; Jin *et al*., 2016).

**Figure 4:**
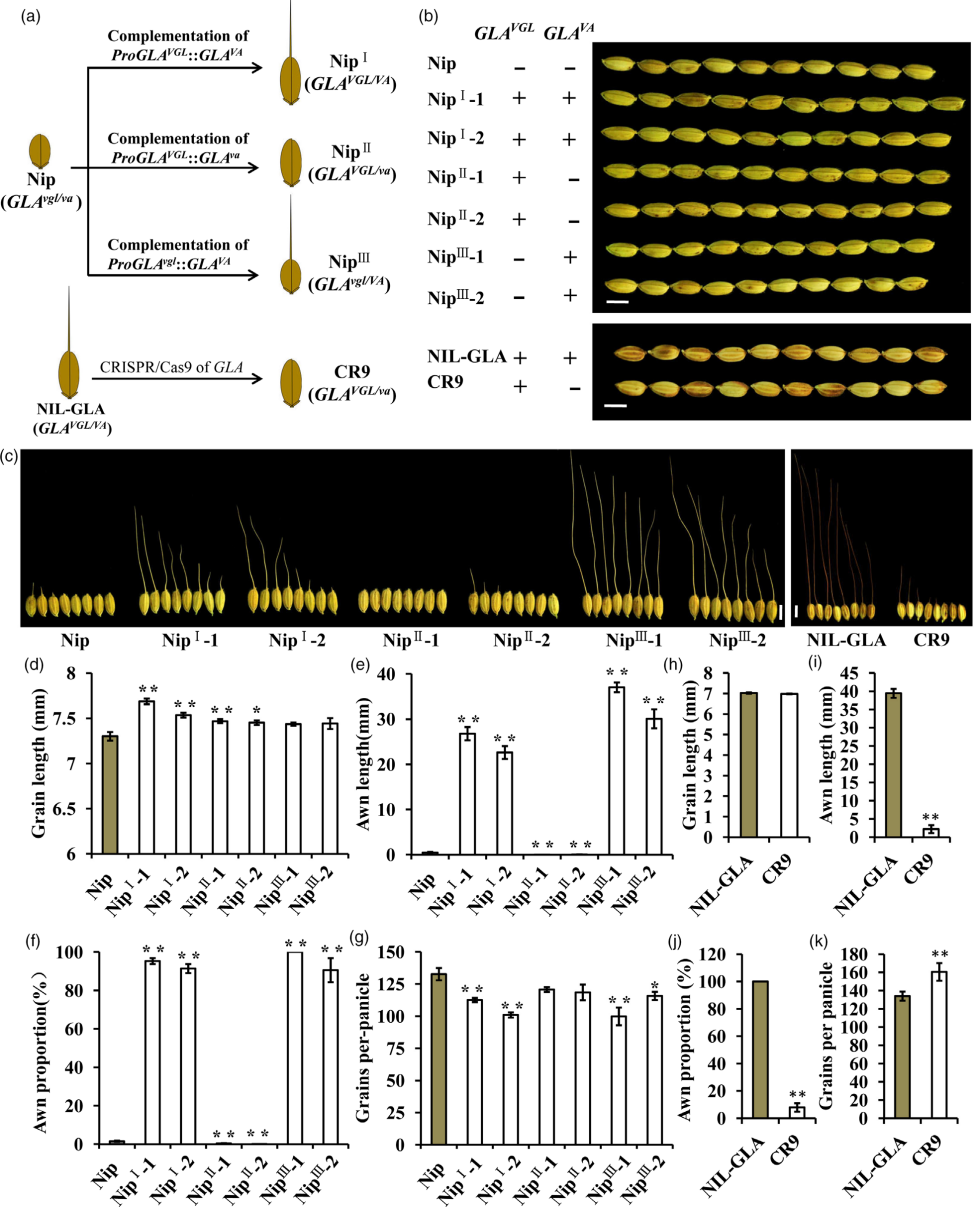
Confirmation of *GLA* in controlling grain length and awn development. (a) Schematic of transgenosis. VGL, variation for grain length. VA, variation for awn development. (b,c) Grain morphology of Nip, NIL‐GLA, six complementation transgenic lines in Nipponbare (Nip) background, and a CRISPR/Cas9 edited line in NIL‐GLA background. Nip^I^‐1 and Nip^I^‐2 are independent transgenic lines containing the *Pro* *GLA* ^*VGL*^ ^:^: *GLA* ^*VA*^ vector. Nip ^II^ ‐1 and Nip ^II^ ‐2 are independent transgenic lines carrying the *Pro* *GLA* ^*VGL*^ :: *GLA* ^*va*^ construct. Nip ^III^ ‐1 and Nip ^III^ ‐2 are independent transgenic lines carrying the *ProGLA* ^*vgl*^:: *GLA* ^*VA*^ construct. CR9 is a CRISPR/Cas9 edited line. ‘+’ and ‘−’ represent functional and non‐functional genotypes. Bar, 5 mm. (d–k) Comparison of grain lengths (d,h), awn lengths (e,i)**,** awn proportions (f,j) and grain numbers per panicle among transgenic lines. Data are represented as means ± SE (*n *=* *15), *P <* *0.05, **P <* *0.01 based on Student's *t*‐tests.

To further confirm the functions of VGL and VA, *ProGLA* ^*VGL*^::*GLA* ^*VA*^, *ProGLA* ^*VGL*^::*GLA* ^*va*^ and *ProGLA* ^*vgl*^::*GLA* ^*VA*^ complementary constructs were introduced into Nip with *GLA* ^*vgl*/*va*^; the transgenic lines were named as Nip^I^, Nip^II^ and Nip^III^ respectively (Figure 4a). Compared to Nip, lines Nip^I^‐1, Nip^I^‐2, Nip^II^‐1 and Nip^II^‐2 showed longer grains, whereas the grain lengths of lines Nip^III^‐1 and Nip^III^‐2 were unchanged (Figure 4b,d). There was no significant change in grain width between transgenic lines (Figure S7c,f). Moreover, the phenotypes for awn length and awn proportion in Nip^I^‐1, Nip^I^‐2, Nip^III^‐1 and Nip^III^‐2 lines were restored (Figures 4c,e and S7a). This further proved that InDel1 and InDel3 in *GLA* were the functional sites for variation in grain length and the awn trait respectively. To further identify the function of *GLA*, a CRISPR‐Cas9 vector (CR) was constructed and transformed into NIL‐GLA, producing a CR9 transgenic line that had no awn and no effect on grain length and width (Figures 4b,c,h–j and S7b,d,e,g). Compared to Nip, lines Nip^I^‐1, Nip^I^‐2, Nip^III^‐1 and Nip^III^‐2 had lower grain number per panicle (Figure 4g). Compared to NIL‐GLA, the grain number per panicle of line CR9 was significantly increased (Figure 4k), thus suggesting that *GLA* ^*va*^ increased grain number per panicle. All of these results collectively verified that the functional variation of *GLA* in regulating grain length was different from that affecting awn development and that natural alleles of *GLA* in the VGL and VA loci might be responsible.

### Allele *GLA* ^*va*^ was selected before *GLA* ^*vgl*^

**Figure 5:**
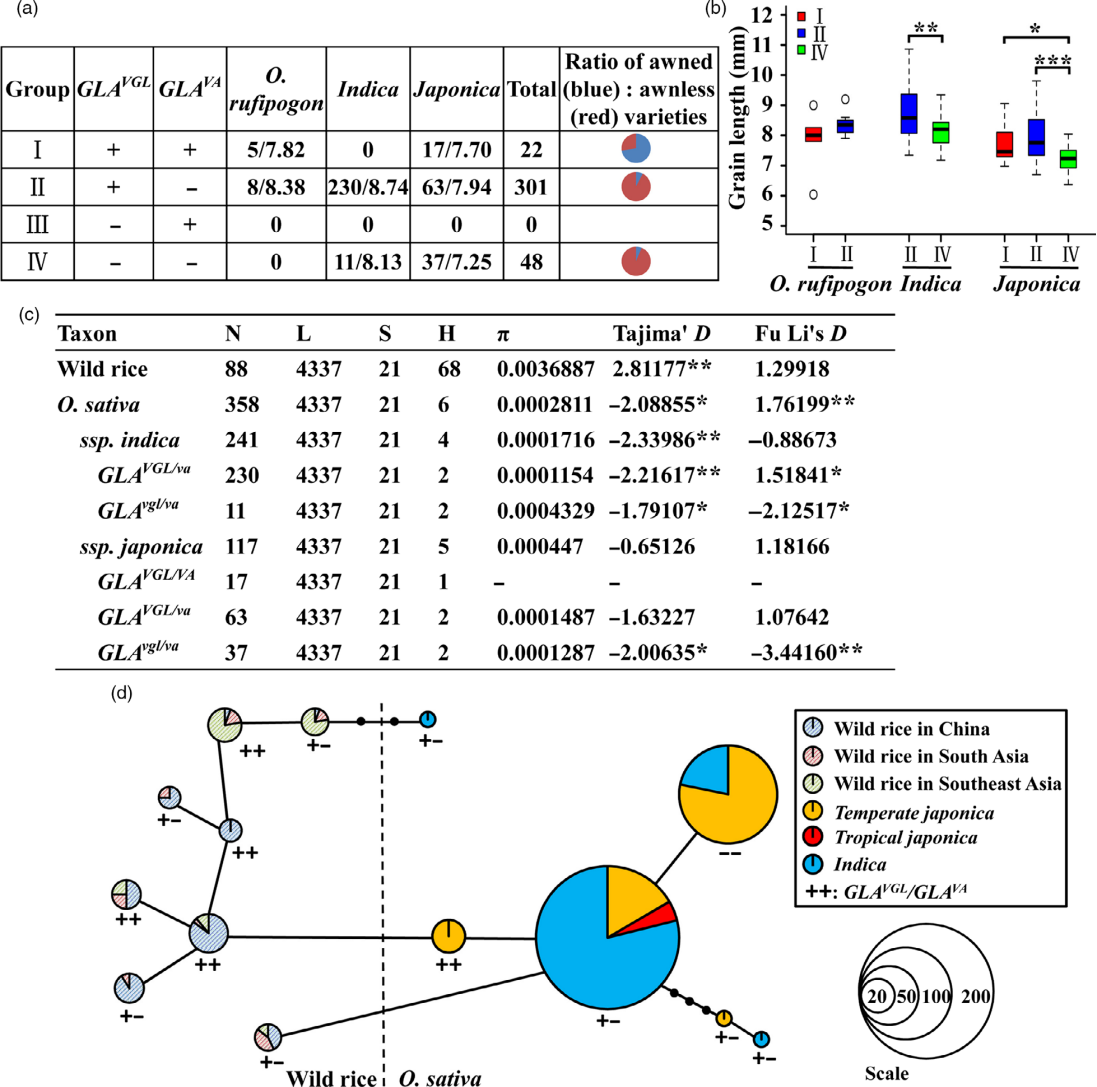
Evolutionary relationships of *GLA* ^*VGL*^ ^*/*^ ^*VA*^ for grain length and awn traits. (a,b) Haplotype analyses of *GLA* ^*VGL*^ and *GLA* ^*VA*^ in 371 rice accessions for grain length and awns. ‘+’ and ‘‐’ represent functional and non‐functional genotypes. (c) Nucleotide variation and neutrality test of *GLA*. N, L, S, H and π represent total number of sequences, average length (bp) of the sequences per taxon, number of polymorphic sites, number of haplotypes and average number of pairwise nucleotide differences per site calculated based on the total number of polymorphic sites respectively. *P <* *0.05, **P <* *0.01. (d) Minimum spanning tree for the *GLA* region based on 358 cultivated and 88 diverse wild rice sequences.

We categorized 371 rice accessions (358 cultivated and 13 wild rice) into four groups according to non‐functional (−) or functional (+) alleles of *GLA* ^*VGL*^ and *GLA* ^*VA*^. There was no accession in group III. Most accessions in group I (five *O. rufipogon*, 11 of 17 in *japonica*) with *GLA* ^*VA*^ had awned phenotypes and most accessions in groups II (53 of 63 in *japonica*, 225 of 230 in *indica*) and IV (11 in *indica*, 34 of 38 in *japonica*) with *GLA* ^*va*^ were awnless (Figure 5a, Data S2). Grain lengths of wild rice accessions with *GLA* ^*VGL*^ showed no significant difference between groups I and II. However, compared to grain lengths of group IV in *japonica* and *indica*, respectively, varieties with *GLA* ^*VGL*^ in group I in *japonica*, group II in *japonica* and group II in *indica* had longer grains (Figure 5b, Data S2). These results indicated that the grain length and awn phenotype of various rice varieties were consistent across different haplotype combinations. Moreover, most cultivated rice accessions belonged to group II with *GLA* ^*VGL/va*^.

*GLA* is considered a domestication gene (Bessho‐Uehara *et al*., 2016; Jin *et al*., 2016) that controls grain length and awn development. The question then was what genotypes of *GLA* ^*VGL/VA*^ were selected artificially. A nucleic acid diversity analysis was conducted and a neutrality test was carried out based on the variant sites (~4.4 kb). The nucleotide diversity of *gla* in cultivated rice was significantly lower than that of *GLA* in wild rice. Tajima's *D* test (*P *<* *0.01) revealed that *GLA* in cultivated and wild rice deviated significantly from neutrality (Figure 5c) indicating that *GLA* in wild rice was subjected to balanced natural selection and that *gla* in cultivated rice had undergone directional artificial selection. Thus selection in wild and cultivated rice led to different phenotypes. Natural selection in wild rice favoured awned phenotypes, whereas artificial selection favoured awnlessness (*GLA* ^*va*^) in *indica* and short grains plus awnlessness (*GLA* ^*vgl/va*^) in *japonica* (Figure 5c).

In order to explore evolutionary relationships between *GLA* ^*VGL*^ and *GLA* ^*VA*^, 358 cultivated and 88 wild rice accessions were used to produce a minimum spanning tree. Wild rice had both *GLA* ^*VGL/VA*^ (documented as ++) and *GLA* ^*VGL/va*^ (+−) genotypes. However, *temperate japonica* rice that differentiated from wild rice in China did not acquire the *GLA* ^*va*^ allele. *Temperate japonica* with *GLA* ^*VGL/VA*^ first produced the natural variant *GLA* ^*va*^. The long grain, awnless genotype *GLA* ^*VGL/va*^ was obtained by artificial selection. Subsequently, the *GLA* ^*VGL/va*^ allele changed to *GLA* ^*vgl/va*^ (− −) in cultivated rice. These results indicated that the *GLA* ^*va*^ allele was selected before *GLA* ^*vgl*^. Moreover, *indica* rice first appeared in a long‐grain, awnless combination and *japonica* and *indica* had different selection histories, leading to short‐grain, awnless and long‐grain, awnless genotypes respectively (Figure 5d). Thus, we hypothesize that *GLA* ^*va*^ domestication occurred before divergence of the *indica* subspecies.

### *An‐1* was preferentially domesticated before *GLA* and *An‐2*

**Figure 6:**
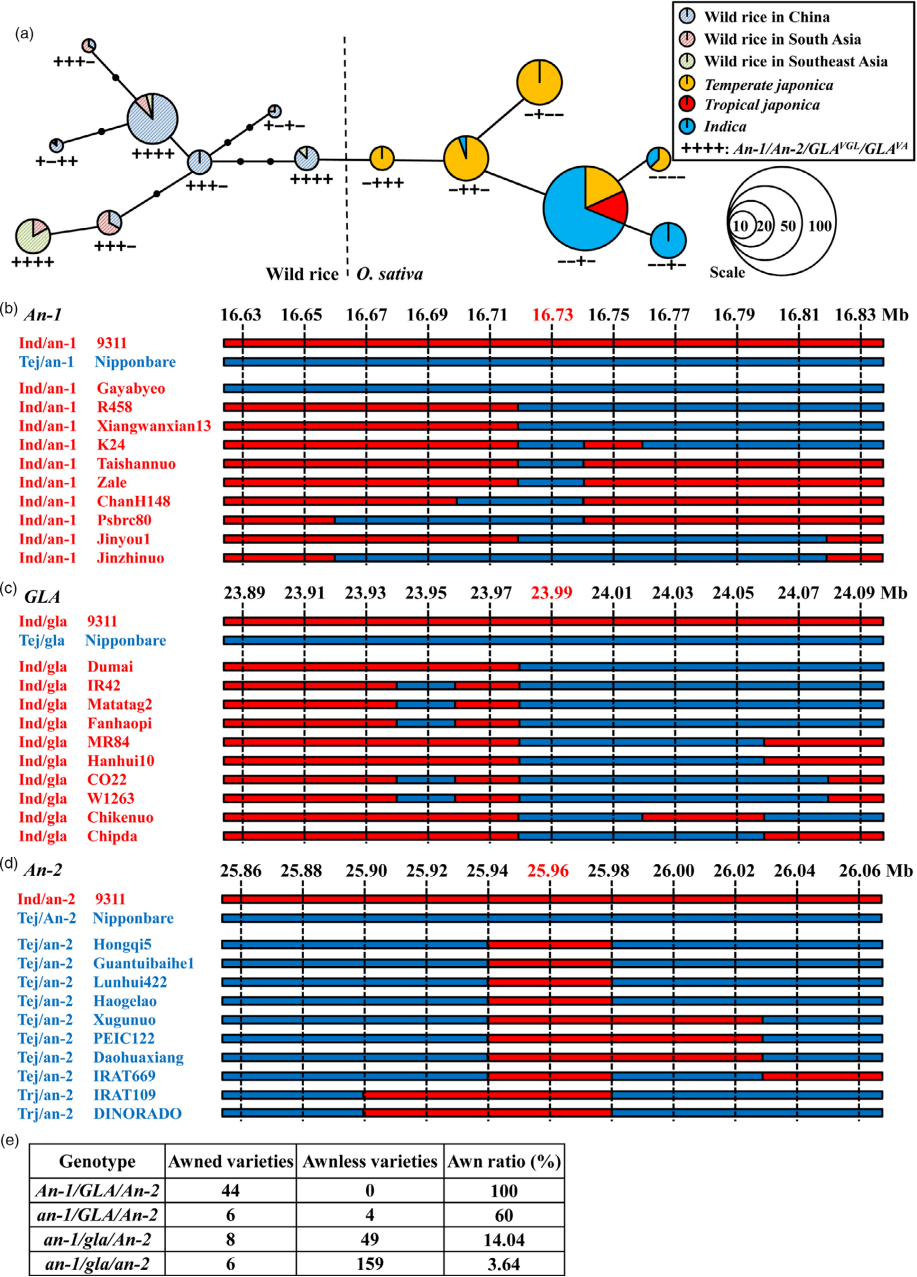
Evolutionary relationships among *An‐1*, *GLA* and *An‐2*. (a) Minimum spanning tree for the *An‐1*, *GLA* and *An‐2* regions among 145 cultivated and 69 wild rice accessions. (b–d) Analyses of introgressed regions for *An‐1* (b), *GLA* (c) and *An‐2* (d). Ten SNP markers in 100 kb intervals upstream and downstream of these genes were used. Ind, *indica*. Tej, *temperate japonica*. Trj, *tropical japonica*. Nipponbare and 9311 were reference sequences. Red and blue bars represent *indica* and *japonica* genotypes respectively. Markers at 16.73, 23.99 and 25.96 Mb were the closest to *An‐1*, *GLA* and *An‐2* respectively. (e) Comparison of rates of contribution to awn traits among *An‐1*,* An‐2* and *GLA*.

The currently cloned genes controlling awn traits are *An‐1*,* An‐2* and *GLA/GAD1/RAE2*. To determine the evolutionary relationships among these genes during domestication a minimum spanning tree was constructed using 145 cultivated and 69 wild rice accessions. The results showed that a change in *An‐1* was the first step in domestication. This produced *temperate japonica* with *an‐1/An‐2/GLA* ^*VGL*^ */GLA* ^*VA*^ (symbolized −+++). Then, *temperate japonica* with *an‐1* was domesticated at the functional position *GLA* ^*VA*^ leading to *GLA* ^*va*^ (−++−). By this time, *indica* rice had evolved. Part of the cultivated population with *an‐1/An‐2/GLA* ^*VGL*^ */GLA* ^*va*^ (−++−) underwent natural variation in *GLA* ^*VGL*^ leading to the *an‐1/An‐2/GLA* ^*vgl*^/*GLA* ^*va*^ (−+−−) genotype. Other cultivated rice (−++−) lines were domesticated by change in *An‐2*, and *an‐1/An‐2/GLA* ^*VGL*^/*GLA* ^*va*^ (–+−) appeared (Figure 6a). Thus *An‐1* was domesticated first, and was subsequently followed by changes in *GLA* and *An‐2*. Variation of *GLA* ^*VGL*^ occurred after the *GLA* ^*VA*^ domestication step.

The minimum spanning tree of *GLA* or three domestication genes controlling awn development clustered *indica* and *japonica* together, similar to previous studies on gene flow (Choi *et al*., 2017; Huang *et al*., 2012). To determine whether these genes were associated with gene flow of domestication alleles we performed analyses of introgressed regions. The *an‐1* and *gla* domestication alleles were transferred from *japonica* to the *indica* population, whereas the *an‐2* allele was transferred from *indica* to *japonica* (Figure 6b–d). These results demonstrated significant gene flow between the *japonica* and *indica* populations.

We then evaluated the contributions of *An‐1*,* An‐2* and *GLA* to awn development. The genotypes and phenotypes of 232 cultivated and 44 wild rice accessions revealed that after domestication of *An‐1*, the frequency of accessions with awns decreased from 100% to 60%, and awn proportion fell to 14.04% and 3.64% after *GLA* and *An‐2* domestication respectively (Figure 6e). We concluded that *An‐1* made the largest contribution to awn phenotype, followed by *GLA* and *An‐2* respectively.

### The selection to *GLA* ^*vgl*^ is beneficial to rice quality improvement

**Figure 7:**
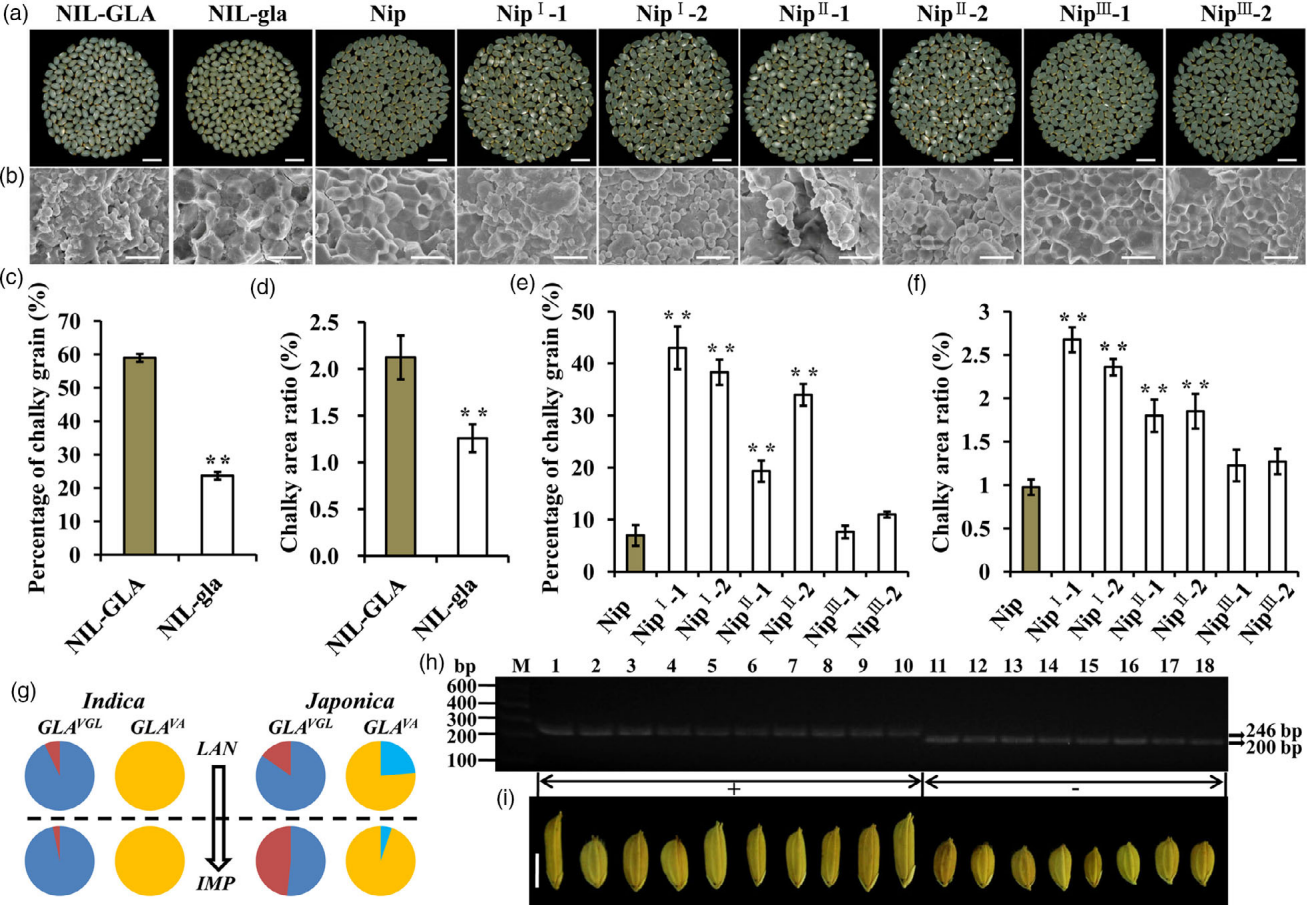
Improved grain quality produced by null *GLA* ^*VGL*^ . (a) Comparison of milled rice in NILs, Nip and complementation lines. Bar, 1 cm. (b) SEM images of starch granules in transverse sections of grains. Bar, 10 μm. (c–f) Percentages of chalky grain (c,e) and chalky area ratio (d,f). Data are represented as means ± SE. (*n *=* *15), *P <* *0.05, **P <* *0.01 based on Student's *t*‐tests. (g) Allele frequency analysis of *GLA* ^*VGL*^ and *GLA* ^*VA*^ . Blue and red colors represent functional ( *GLA* ^*VGL*^ ) and non‐functional (*GLA* ^*vgl*^) alleles of the VGL locus. Skyblue and orange colors represent functional ( *GLA* ^*VA*^ ) and non‐functional (*GLA* ^*va*^) alleles of the VA locus. Pie charts above and below the horizontal line represent landraces (LAN) and improved varieties (IMP). (h) Genotyping analyses of *GLA* ^*VGL*^ in 18 diverse accessions of cultivated rice. We used a pair of primers (Table S2) to amplify gene segments containing the variable InDel1 site. The length of the PCR product amplified from accessions with *GLA* ^*VGL*^ (+) was 246 bp and that from accessions with *GLA* ^*vgl*^ (−) was 200 bp. (i) Grains of the 18 cultivated rice accessions. Bar, 5 mm. *P <* *0.05, **P <* *0.01, ***P <* *0.001 based on Student's *t*‐tests.

Grain length is closely related to grain quality (Zhao *et al*., 2018). We evaluated the grain quality of NILs and found that rice milled from NIL‐GLA had high percentages of chalky grain and chalky area with loose and irregular starch granules, unlike that from NIL‐gla, which displayed compactly arranged and largely sharp‐edged polygonal starch granules (Figure 7a–d). These results indicated that *GLA* caused poor quality. The grain qualities of transgenic plants were also investigated. Compared to Nip, Nip^I^‐1, Nip^I^‐2, Nip^II^‐1 and Nip^II^‐2, transgenic plants had a higher proportion of chalky grain and small, messy starch granules, whereas Nip, the Nip^III^‐1 and Nip^III^‐2 lines had similar phenotypes (Figure 7a,b,e,f). Thus *GLA* ^*VGL*^ caused poor quality whereas *GLA* ^*VA*^ had no influence on rice grain quality.

Given that *GLA* ^*VGL*^ and *GLA* ^*VA*^ affected grain length, grain quality and awn development the question was how to make use of *GLA* in genetic improvement. We surveyed the functional allelic frequencies of *GLA* ^*VGL/VA*^ in the panel of 358 varieties. Compared with landrace groups (LAN), the non‐functional allele frequencies of *GLA* ^*vgl*^ and *GLA* ^*va*^ in improved accessions (IMP) were significantly increased in *japonica*, unlike *indica* in which the functional allele *GLA* ^*VA*^ did not exist and the functional allele frequency of *GLA* ^*VGL*^ was increased (Figure 7g). These results implied that *indica* and *japonica* had different selection histories during genetic improvement. The *GLA* ^*VGL*^ allele for long grain phenotype was enriched in *indica* with *GLA* ^*va*^, whereas the *GLA* ^*vgl/va*^ allele for good quality, awnlessness and short grain was utilized in *japonica*. Additionally, a functional molecular marker (J2) for the VGL locus was developed (Data S1). Genotyping analyses using the J2 marker in 18 cultivars further confirmed that *GLA* ^*VGL*^ was most likely responsible for long‐grain phenotype (Figure 7h,i, Data S6). The J2 marker should be applied in future molecular marker assisted breeding.

## Discussion

### *GLA* has pleiotropic effects on grain length, awn development, grain quality and grain number

Wild rice exhibits long, barbed awns that prevent granivore predation, but is detrimental for harvesting and post‐harvest processing. Hence awnless varieties were produced during domestication of wild rice (Kovach *et al*., 2007; Sweeney and McCouch, 2007). Several genes associated with awn development have been identified. However, various genes for yield‐related traits, such as grain length, grain number and yield per plant were often closely associated with awn length and hence were neglected because of the selective sweep of awn traits. Thus, the molecular mechanisms of these genes needed to be investigated individually.

In this study, we identified *Os08 g37890* as *GLA*, a new allele of *RAE2*/*GAD1*. *GLA* exhibited pleiotropic effects on grain length, grain quality, grain number and awn development, and had different functional sites. Our study identified a new functional site, *GLA* ^*VGL*^, that regulated grain length and grain quality and further verified the *GLA* ^*VA*^ functional site controlling awn development. In addition, *GLA* ^*VGL*^ affected grain length at the transcriptional level, consistent with RNAi results in *GAD1* studies (Jin *et al*., 2016). During genetic improvement, *japonica* rice underwent improved quality, shorter grain and awnlessness, whereas *indica* became long grained and awnless. A functional molecular marker developed for *GLA* ^*VGL*^ can be used in breeding programs. On the basis of the long‐grain, awnless phenotype in *indica* and short‐grain, awnless traits in *japonica*, grain quality and grain length can be further improved.

### Origin and evolution of awn‐associated domestication loci

*An‐1* influences the formation of awn primordia by regulating cell division (Luo *et al*., 2013). *An‐2* induces awn elongation by increasing the cytokinin concentration in awn primordial (Gu *et al*., 2015; Hua *et al*., 2015). *GAD1/RAE2*, an EPFL family member, regulates awn length (Bessho‐Uehara *et al*., 2016; Jin *et al*., 2016). Evidence from this study indicated that *An‐1* was the first of these genes to undergo domestication, followed by *GLA* and then *An‐2*. This also suggested that *An‐1* is the most important gene controlling awn development. Minimal spanning tree and introgression analyses implied that *An‐1* and *GLA* might have been domesticated preferentially in *japonica* before the divergence of *indica*, but subsequently, the *An‐2* allele was transferred from *indica* to *japonica*. Hence, some questions are worth pondering. Firstly, given that *An‐2* makes the least contribution to awn formation why did introgression of *An‐2* actually occur. It may be that *An‐2* was linked to other genes and that transfer was due to linkage drag. The next issue is whether awn–related domestication genes arose only once and were dispersed by introgressive hybridization or whether they arose more than once. Several domestication‐related loci, such as *OsLG3b*,* Ghd7*,* LG1* and *LABA1*, show introgression signals between the subspecies, but others (*Sh4*,* qSH3*,* qSH1*,* Prog1*, and *Rc*) lack such signals (Civan and Brown, 2018; Yu *et al*., 2018).

The origin of cultivated rice is a continuing, controversial issue with variable lines of evidence for single and multiple origins. Multiple origins are currently supported by most academics (Choi *et al*., 2017; Londo *et al*., 2006; Sun *et al*., 2018). Therefore, by combing previous studies and our results, we speculate that proto*‐japonica* from wild rice in Southern China went through the *An‐1* and *GLA* domestication steps prior to *japonica/indica* differentiation and produced domesticated proto*‐japonica* varieties with the *an‐1/gla* genotype. After emergence of proto*‐indica* varieties from *O. rufipogon* in Southeast and South Asia they underwent domestication of *An‐2*. Later, populations of proto*‐japonica* with *an‐1/gla* and proto*‐indica* with *an‐2* merged with each other and hybridization led to the current *japonica* and *indica* forms with genotype *an‐1/gla/an‐2* (Figure S8). Nevertheless, the detailed evolutionary relationships based on awn traits need to be further analysed. Nonetheless, discovery of the new *GLA* allele will be of value for understanding the domestication and genetic improvement of rice.

## Experimental procedures

### Plant materials

Mapping populations were constructed from a cross between *japonica* cv. SYL (awned and long grains) as donor parent and *japonica* cv. Nipponbare (Nip, awnless and short grains) as recurrent parent. Recessive awnless and dominant awned individuals in the BC_3_F_4_ generation with short and long grains respectively, were chosen as near isogenic lines (NILs), named NIL‐gla and NIL‐GLA. A segregating BC_3_F_3_ population containing 203 individuals was used for preliminary mapping of *GLA*. A BC_3_F_4_ population with 12 204 individuals was used for fine mapping. Plant materials were grown under natural paddy conditions at Beijing, or at Sanya in Hainan province.

### Fine mapping and candidate gene analysis

Four hundred and seventy‐seven SSR markers distributed across all 12 rice chromosomes were screened in a bulked segregant analysis (BSA) to identify the linked markers associated with the awn trait (Zhang *et al*., 1994) and were also used for preliminary mapping. Sequence‐tagged site (STS) markers were then developed for fine mapping using DNASTAR software.

Candidate gene annotations were made on the basis of the Rice Annotation Project Database (http://rice.plantbiology.msu.edu/index.shtml). Genomic DNA fragments of four predicted genes were amplified from the DNA of NIL‐gla and NIL‐GLA and sequenced, respectively. Sequences were analysed using the SeqMan program in DNASTAR software. Primers are listed in Data S1.

### cDNA and quantitative RT‐PCR

Total RNA was extracted from 5 cm panicles of NIL‐GLA and NIL‐gla using Trizol reagent (Invitrogen, Carlsbad, USA). To eliminate contamination by genomic DNA 50 μg of RNA was digested with Recombinant DNase I (RNase‐free) (Takara, Japan) as described by the manufacturer. Two microgram of DNaseI‐treated RNA was reversely transcribed using M‐MLV Reverse Transcriptase (Takara) with an oligo (dT18) primer. The reaction product was diluted three times as template. Quantitative RT‐PCR (qRT‐PCR) was carried out as previously reported (Sun *et al*., 2018). *OsActin1* was used for internal reference (Data S1).

### Vector construction and rice transformation

To make the genomic DNA complementation construct (*ProGLA* ^*VGL*^::*GLA* ^*VA*^) containing functional InDel1 and InDel3 a 4717 bp genomic DNA fragment of *GLA* harbouring a 3292 bp region upstream of the initiation codon and a 919 bp region downstream of the start codon was amplified from NIL‐GLA using primers LA and cloned into the *PmeI* and *SacI* sites of the binary plant expression vector pMDC163 (Curtis and Grossniklaus, 2003). Similarly, *ProGLA* ^*VGL*^::*GLA* ^*va*^ and *ProGLA* ^*vgl*^::*GLA* ^*VA*^ vectors were constructed using primers LA/la and la/LA respectively. The *ProGLA* ^*VGL*^::*GLA* ^*va*^ vector contained a 3292 bp corresponding region upstream of the initiation codon of *GLA* from NIL‐GLA line and a 1421 bp region downstream of the initiation codon of *GLA* from NIL‐gla line. The *ProGLA* ^*vgl*^::*GLA* ^*VA*^ vector consisted of a 3241 bp region upstream of the initiation codon of *GLA* from NIL‐gla and a 1425 bp region downstream of the start codon of *GLA* from NIL‐GLA. A CRISPR‐Cas9 construct (CR) targeting the 74th–93th nt (5′‐ GCTGCTACAGCAAGTGCTAC‐3′) of the second exon of *GLA* was made as previously described (Feng *et al*., 2013; Mao *et al*., 2013; Zhang *et al*., 2014). To generate transgenic plants, the constructs were introduced into *Agrobacterium tumefaciens* strain EHA105 and subsequently transformed into Nipponbare (Nip) or NIL‐GLA by Agrobacterium‐mediated transformation (Hiei *et al*., 1994).

### Phenotypic evaluation

Three main panicles of each plant were collected for analysis of awn length (>1 mm), awn proportion and grains per panicle. The awn length of the panicle was represented by the average of the apical spikelet on each primary branch. Awn proportion was estimated as the number of awned spikelets per panicle. One hundred fully mature grains were randomly chosen for measurement of grain length and width. The grains were placed on a scanner for scanning. Grain length and width were calculated by analysing the scanned images (Luo *et al*., 2013). Grain weight per plant was represented by the dry weight of all grains from one plant. At least 15 individuals were used for phenotyping of NIL‐GLA, NIL‐gla and Nip transgenic plants.

### Scanning electron microscopy

Young spikelets from NIL‐GLA and NIL‐gla were fixed in 2.5% glutaraldehyde‐phosphate buffer saline fixative solution and dehydrated through an ethanol series (30, 50, 70, 80, 90, 95 and 100%). After dehydration the samples were dried with a carbon dioxide critical‐point dryer. Mature grains were cleaned with 1% Tween 20 and dried in a 45 °C oven. Dried grains and spikelets were gold plated and observed using a Hitachi S‐2460 scanning electron microscope at 15 kV. Cell number, cell length and cell width of lemmas were quantified from scanning electron microscopy images. Quantification was represented by the average of 15 grains.

### Transient expression assays of promoter activity

To make constructs *ProGLA* and *Progla*,* GLA* promoters with and without InDel1 were amplified using primers TEGLA and TEgla respectively (Data S1) and cloned into the *PmeI* and *SacI* sites of the pMDC162 vector. *ProGLA*,* Progla* and *Pro35S::LUC* plasmid used as an internal control were co‐transferred into tobacco leaves. The ratios of GUS/LUC (luciferase) were used as relative promoter activities (Sun *et al*., 2018; Zhang *et al*., 2017). Three biological repeats, each with four technical replicates were analysed for each vector.

### Sub‐cellular localization of *GLA*

*GLA* and *gla* cDNAs were amplified and cloned into *pSuper1300* vector, containing a Super promoter (Ni *et al*., 1995; Yang *et al*., 2010), to obtain *ProSuper::GLA‐GFP* and *ProSuper::gla‐GFP* constructs respectively. The resulting vectors and CD3‐1007, a plasma membrane marker (Nelson *et al*., 2007) were co‐transfected into rice protoplasts. After culturing for 16 h at 28 °C fluorescence signals were observed as described (Zhang *et al*., 2011).

### *GLA*‐based association analysis and haplotype analysis

A worldwide set of 358 cultivated rice accessions selected from the 3K‐Rice Project (Rice Functional Genomics and Breeding database, RFGB) (Zheng *et al*., 2015) and 13 Asian wild rice accessions provided by the National Germplasm Nanning Wild Rice Nursery were used for the study (Data S2). Genomic DNA fragments of *GLA* from different accessions were amplified using the V1, V2, V3 and V4 primers (Data S1 and S2)**,** sequenced and analysed by DNASTAR software. Variable sites, including 33 SNPs (single nucleotide polymorphisms) and 13 InDels (insertion/deletion polymorphisms), were used for *GLA*‐based association analysis (Liu *et al*., 2016; Wang *et al*., 2016; Xiong *et al*., 2018). Significant sites based on the –log_10_ (*P*) values were used for haplotype analyses (Data S2). The InDel1 and InDel3 alleles of *GLA* in 371 rice accessions (Data S2) were analysed from the sequencing results and then subjected to genotyping. Group analyses were carried out as previously reported (Sun *et al*., 2018).

### Allelic frequencies and neutrality tests

The 358 accessions (143 landraces and 215 improved accessions; Data S2 and S5) were analysed for the *An‐1*,* An‐2* and *GLA* functional loci controlling grain length or awn development and genotyped as non‐functional or functional alleles (Sun *et al*., 2018).

Eighty eight wild rice accessions provided by Dr Song Ge and 358 cultivars (241 *indica* and 117 *japonica*) varieties were used to evaluate nucleic acid diversity (π) and neutrality (Tajima's *D* values) of *GLA* (Data S4). InDels and SNPs in *GLA* were identified. The data were processed by DnaSP 5.10 software (Librado and Rozas, 2009).

### Analyses of minimum spanning trees

Rich sequence diversity for *An‐1*,* An‐2* and *GLA* was present in both wild and cultivated rice (Data S5) and some haplotypes were represented by only one accession. We chose 9, 3 and 15 variable sites in *An‐1 An‐2* and *GLA* respectively, and integrated haplotypes containing single varieties in developing the minimum spanning tree (Sun *et al*., 2018). The 358 cultivated and 88 wild rice accessions were used for analysis of the *GLA* region; and 145 cultivated varieties and 69 wild rice accessions were used to determine evolutionary relationships among the three genes. A minimum spanning tree was generated following a previous procedure (Liu *et al*., 2015; Yu *et al*., 2018).

## Competing interests

The authors declare that they have no competing interests.

## Author contribution

ZL, ZZ, HZ, JL and XS conceived the experiment; BL and CC produced the genetic populations; YZ and BL performed experiments; YZ, XZ, YP and XS conducted the phenotypings; YZ, XZ, XS and HG analysed the data; YZ, XZ, HG, ZZ and ZL wrote and revised the manuscript.

## Supporting information


**Figure S1** Co‐segregation of awn and long grain traits.
**Figure S2** Comparison of awn length and awn proportion between NIL‐GLA and NIL‐gla NILs.
**Figure S3** *GLA* genomic sequences in NIL‐GLA and NIL‐gla.
**Figure S4** Sequence alignment of GLA proteins of NIL‐GLA and NIL‐gla.
**Figure S5** Comparison of the amino acid sequences in *GLA* alleles.
**Figure S6** Sub‐cellular localization of GLA protein in rice protoplasts.
**Figure S7** Phenotypic analysis of transgenic plants.
**Figure S8** Proposed evolutionary pathway of awns in *O. sativa*.
**Table S1** Genetic analysis of BC_3_F_3_ and BC_3_F_4_ populations.
**Table S2** Putative genes in the 26.32 kb *GLA* region.

**Data S1** Primers used in this study.
**Data S2** Information of cultivated and wild rice.
**Data S3** Information of cultivated rice used in the correlation analysis between *GLA* mRNA levels and grain length.
**Data S4** Information of cultivated rice and wild rice used in *GLA* analyses of nucleic acid diversity, neutral test and a minimum spanning tree.
**Data S5** Information of cultivated rice and wild rice used in the phylogenetic tree and allele frequency analyses.
**Data S6** Information of cultivated rice used in the genotyping analysis.
