# Supplementary material for: Natural alleles of GLA for grain length and awn development were differently domesticated in rice subspecies japonica and indica
Source: Plant Biotechnol J. 2019 Mar 9;17(8):1547–59. doi: 10.1111/pbi.13080 (PMC6662114; doi:10.1111/pbi.13080)
Supplement: Supplementary file 1 — Figure S1 Co‐segregation of awn and long grain traits. Figure S2 Comparison of awn length and awn proportion between NIL‐GLA and NIL‐gla NILs. Figure S3 GLA genomic sequences in NIL‐GLA and NIL‐gla. Figure S4 Sequence alignment of GLA proteins of NIL‐GLA and NIL‐gla. Figure S5 Comparison of the amino acid sequences in GLA alleles. Figure S6 Sub‐cellular localization of GLA protein in rice protoplasts. Figure S7 Phenotypic analysis of transgenic plants. Figure S8 Proposed evolutionary pathway of awns in O. sativa. Table S1 Genetic analysis of BC3F3 and BC3F4 populations. Table S2 Putative genes in the 26.32 kb GLA region. [file PBI-17-1547-s002.docx]

**Supplemental Information**

**Table S1.** Genetic analysis of BC_3_F_3_ and BC_3_F_4_ populations

**Table S2.** Putative genes in the 26.32 kb *GLA* region

**Figure S1.** Cosegregation of awn and long grain traits

**Figure S2.** Comparison of awn length and awn proportion between NIL-GLA and NIL-gla NILs

**Figure S3.** *GLA* genomic sequences in NIL-GLA and NIL-gla

**Figure S4.** Sequence alignment of GLA proteins of NIL-GLA and NIL-gla

**Figure S5.** Comparison of the amino acid sequences in *GLA* alleles

**Figure S6.** Sub-cellular localization of GLA protein in rice protoplasts

**Figure S7.** Phenotypic analysis of transgenic plants

**Figure S8.** Proposed evolutionary pathway of awns in *O. sativa*

**Supplemental data set1.** Primers used in this study

**Supplemental Data Set 2.** Information of cultivated and wild rice

**Supplemental Data Set 3.** Information of cultivated rice used in the correlation analysis between *GLA* mRNA levels and grain length

**Supplemental data set 4.** Information of cultivated rice and wild rice used in *GLA* analyses of nucleic acid diversity, neutral test and a minimum spanning tree

**Supplemental data set 5.** Information of cultivated rice and wild rice used in the phylogenetic tree and allele frequency analyses

**Supplemental Data Set 6.** Information of cultivated rice used in the genotyping analysis

**(Supplemental Data Set 1-6** are provided in the separate Excel files**)**

**Table S1. Genetic analysis of BC_3_F_3_ and BC_3_F_4_ populations**

| Population | No. of awned plants | No. of awnless plants | χ^2^_3:1_ | P value |
| --- | --- | --- | --- | --- |
| BC_3_F_3_ | 149 | 54 | 0.28 | p>0.5 |
| BC_3_F_4_ | 9,223 | 2,981 | 2.14 | p>0.1 |

**Table S2. Putative genes in the 26.32 kb *GLA* region**

| ORF | Type and putative protein function | Location (bp) | Gene length (bp) |
| --- | --- | --- | --- |
| *LOC_Os08g37850* | Transposon protein, putative, expressed | 23976824—23979650 | 882 |
| *LOC_Os08g37860* | Expressed protein | 23982650—23985965 | 804 |
| *LOC_Os08g37874* | 2-nitropropane dioxygenase family, putative, expressed | 23987970—23996461 | 993 |
| *LOC_Os08g37890* | Secreted signal peptide of the EPIDERMAL PATTERNING FACTOR-LIKE family | 23998787—24000227 | 351 |

All data are from [http://rice.plantbiology.msu.edu/cgi-bin/gbrowse/rice/](http://rice.plantbiology.msu.edu/cgi-bin/gbrowse/rice/%20)

**
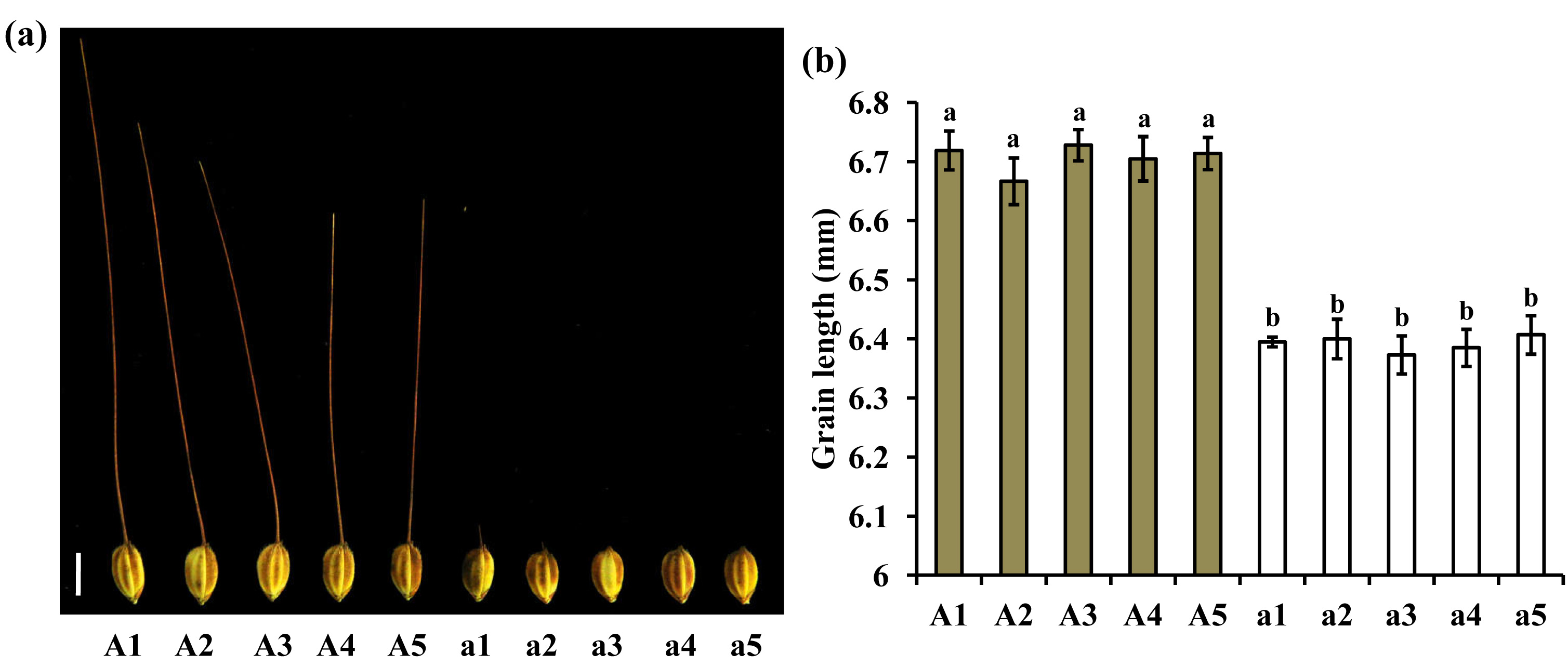
Figure S1. Cosegregation of awn and long grain traits.** (a) Awn phenotypes of five (dominant) awned and five (recessive) awnless BC_3_F_4_ individuals. Bar, 5 mm. (b) Comparison of grain lengths between awned and awnless BC_3_F_4_ individuals. A1 - A5, grains from BC_3_F_4_ awned individuals, a1 - a5, grains from BC_3_F_4_ awnless individuals. Data are means ± s.e. (n = 100 grains, and 3 panicles).


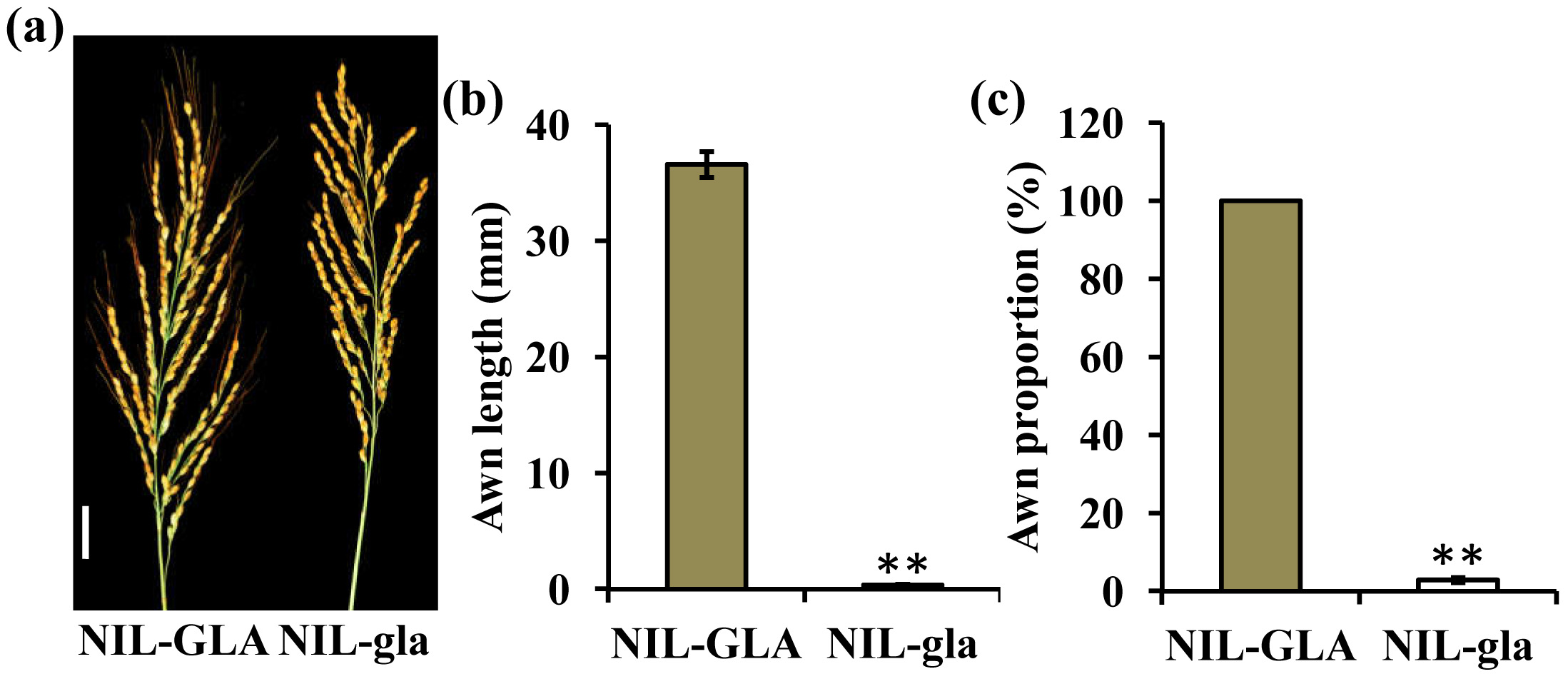


**Figure S2. Comparison of awn length and awn proportion between NIL-GLA and NIL-gla NILs.** (a) Panicles of NIL-GLA (left) and NIL-gla (right). Bar, 2 cm. (b-c) Comparison of awn length (b) and awn proportion (c) between NIL-GLA and NIL-gla NIL lines. Data are means ± s.e. (n = 15), ** *P* < 0.01, based on Student’s *t*-tests.

**
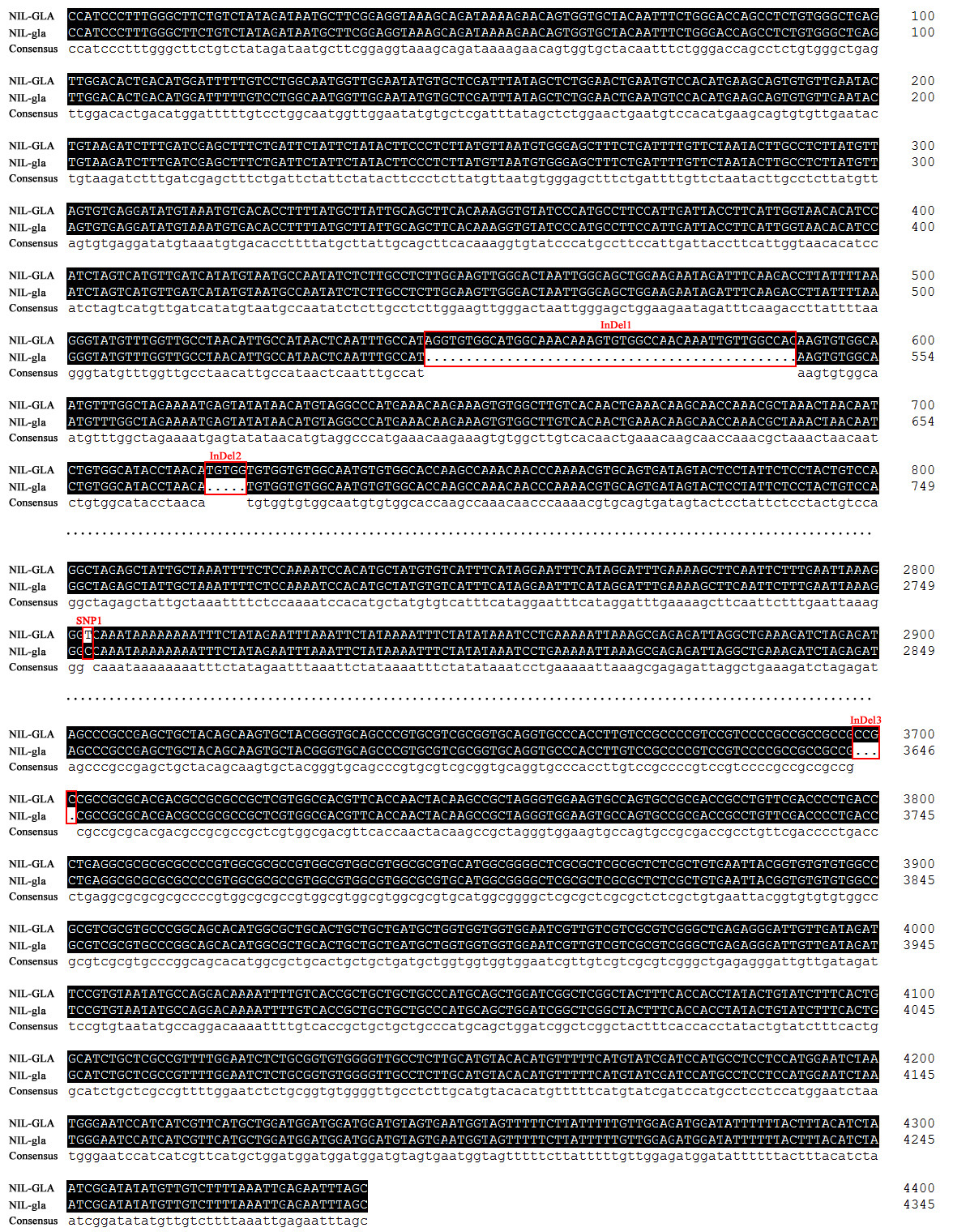
**

**Figure S3.** ***GLA* genomic sequences in NIL-GLA and NIL-gla.** Sequence analysis was conducted by the Megalign program in the DNASTAR software package. Red boxed region indicates differing sequences.


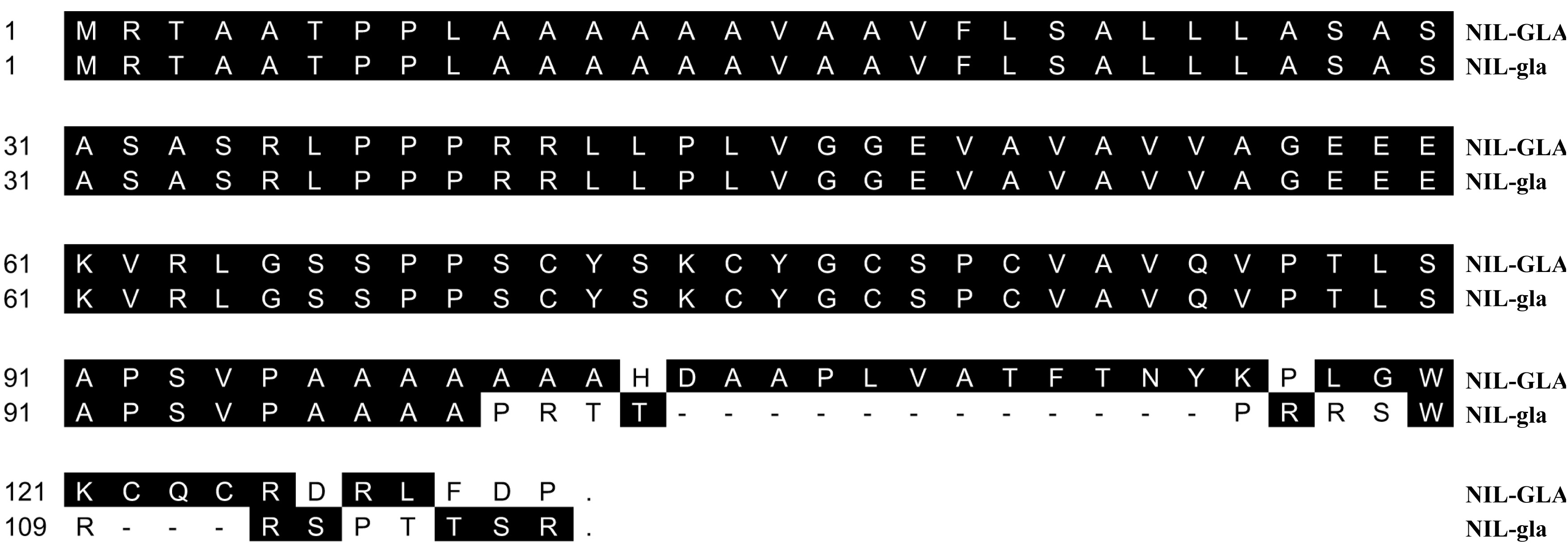
**Figure S4.** **Sequence alignment of GLA proteins of NIL-GLA and NIL-gla.**

**
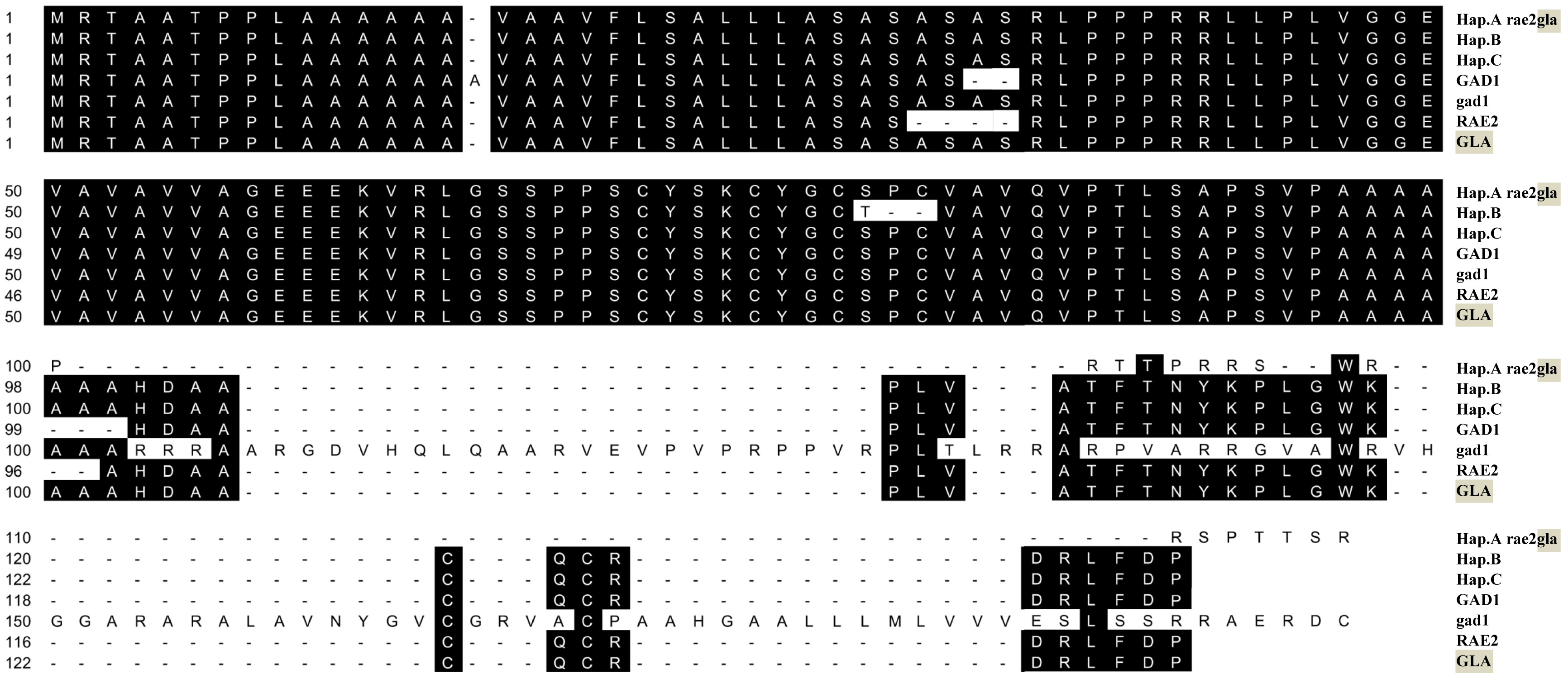
Figure S5. Comparison of the amino acid sequences in *GLA* alleles.**

**
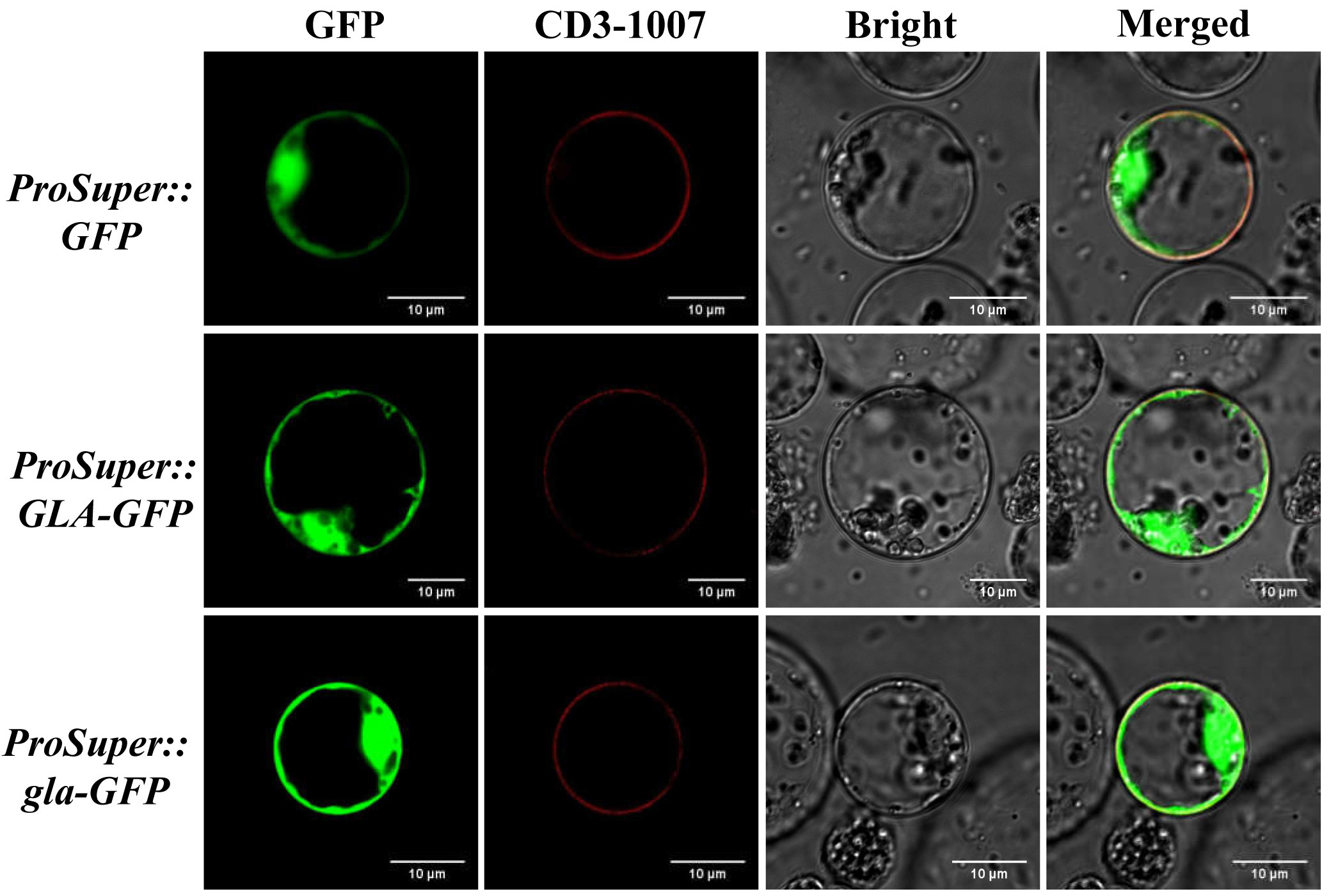
**

**Figure S6. Sub-cellular localization of GLA protein in rice protoplasts.** *ProSuper::GFP* (*pSuper1300*) vector was used as control. Bar, 10 μm.


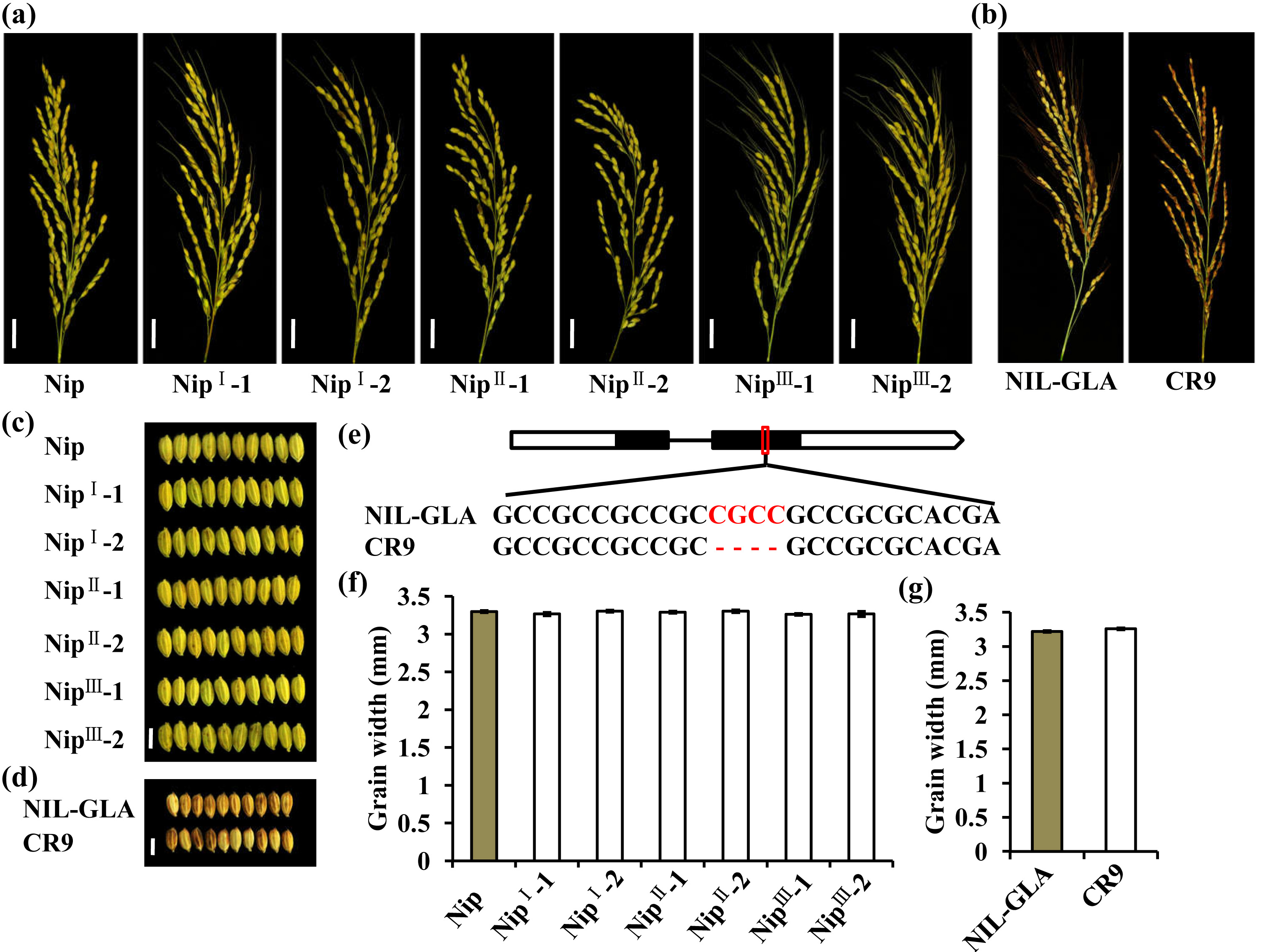


**Figure S7. Phenotypic analysis of transgenic plants.** Panicle (a-b) and grain width (c-d, f-g) comparisons of Nip, NIL-GLA and transgenic plants. Bars, 2 cm, 2 cm, 5 mm and 5 mm, respectively. (e) Gene structure of CR9. White boxes represent 5′ and 3′ UTR regions. Black boxes represent exons. Red box indicates the mutation site in CR9. Data are means ± s.e. (n = 15). Data were compared by Student’s *t*-tests.


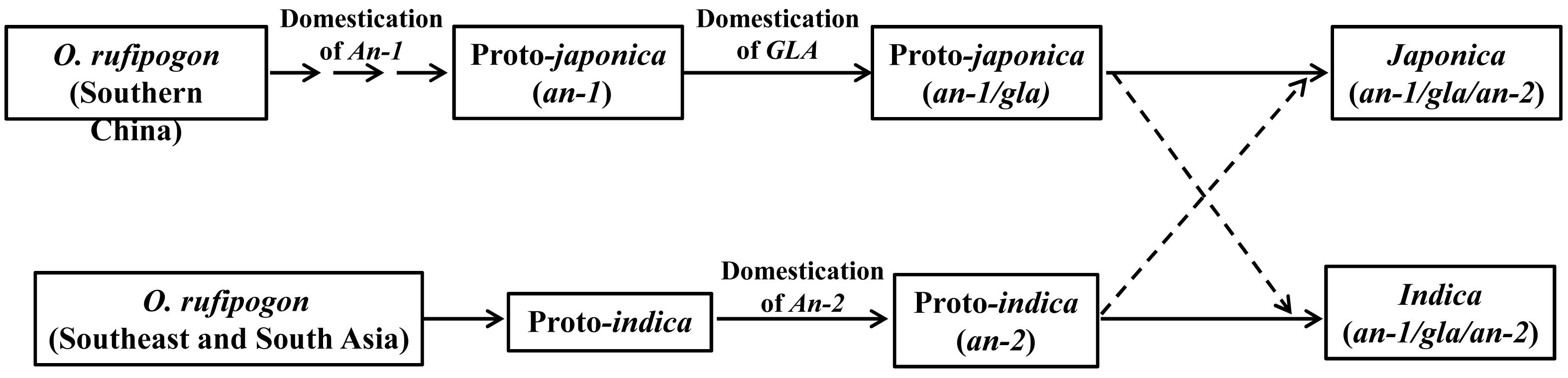


**Figure S8.** **Proposed evolutionary pathway of awns in *O. sativa*.** Black dotted lines with arrows represent gene introgression.
